# The risk factors of postoperative delirium in patients with hip fracture: implication for clinical management

**DOI:** 10.1186/s12891-021-04091-1

**Published:** 2021-03-07

**Authors:** Weifang Xu, Haiping Ma, Wang Li, Chen Zhang

**Affiliations:** 1 Department of Anesthesiology, The First affiliated hospital of XinJiang Medical University, Urumqi, China; 2 School of Public Health, Xinjiang Medical University, No.393 Xinyi Road, Xinjiang 830054 Urumqi, PR China; 3 Key Laboratory of Xinjiang Metabolic Disease, Clinical Medical Research Institute, The First Affiliated hospital of XinJiang Medical University, Urumqi, PR China

**Keywords:** Delirium, Hip fracture, Risk, Surgery, Nursing care

## Abstract

**Background:**

Delirium is a common complication of hip surgery patients. It is necessary to investigate the epidemiological characteristics and related risk factors of delirium after hip fracture surgery, to provide evidence supports for the prevention and management of delirium.

**Methods:**

Hip fracture patients admitted to our hospital for surgical treatment from March 2018 to March 2020 were identified as participants. The characteristics and laboratory examinations in patients with and without postoperative delirium were compared and analyzed. Logistic regression analyses were conducted to ascertain the independent risk factors, and the area under the curve (AUC) were calculated to analyze the predictive value.

**Results:**

A total of 568 postoperative patients with hip fracture were included, the incidence of delirium in postoperative patients with hip fracture was 14.44 %. The preoperative albumin (OR 4.382, 2.501 ~ 5.538), history of delirium (OR 2.197, 1.094 ~ 3.253), TSH (OR1.245, 1.077 ~ 1.638), the resting score on the first postoperative day (OR1.235, 0.944 ~ 1.506) and age(OR1.185, 0.065 ~ 1.814) were the independent risk factors for the postoperative delirium in patients with hip fracture(all p < 0.05). The AUC of albumin, history of delirium, TSH, the resting score on the first postoperative day and age were 0.794, 0.754, 0.746, 0.721 and 0.689 respectively.

**Conclusions:**

The incidence of delirium in postoperative patients with hip fracture is rather high, especially for patients with old age and history of delirium. Monitoring albumin, TSH and resting score may be beneficial to the management of postoperative delirium.

## Background

Hip fracture is a very common kind of disease in clinically elderly patients. According to reports, by 2050, the number of patients with hip fractures each year around the world is about 4.5 million[1, 2]. The incidence of hip fractures in the elderly has increased year by year, with female patients accounting for 75 % of all hip fracture patients[3]. This has also brought great challenges and pressures to clinical medical workers throughout world. How to treat and care for these patients correctly and effectively has become a top priority for clinical research. Surgical treatment has become the first choice for the treatment of hip fracture patients[4]. But at the same time, surgical treatment has also brought a series of postoperative complications, which needs to be highly valued and prevented by us.

Delirium is a very common postoperative complication in elderly patients with hip fractures[5]. Its main manifestations are unconsciousness, difficulty concentrating, disorder of perception and thinking and disturbance of sleep[6]. Among elderly hip fracture patients, the incidence of delirium is 20–50 %[7]. Delirium may cause huge adverse impact on patients, which may extend the postoperative recovery of patients, increase the medical expense and burden, and prolong the length of hospital stay, and even increase the mortality[8]. Therefore, the prevention and treatment of postoperative delirium are crucial to the prognosis of patients with hip fracture.

There are several studies[9–11] on the possible risk factors for postoperative delirium in elderly hip fracture patients, but the results remain inconsistent or even conflicting. There are many possible risk factors reported, including age, education background, alcohol consumption, anesthesia and blood loss etc. However, the independent risk factors for the postoperative are not yet clear, which needs further verifications. Therefore, we attempted to conduct this retrospective study to identify the potentially relevant risk factors for the postoperative delirium in patients with hip fracture.

## Methods

### Ethical concerns

This study has been approved by the Ethics Committee of our Hospital (approval number: 201800387-2a), all patients signed the written informed consent.

### Patients

The hip fracture patients admitted to our hospital for surgical treatment from March 2018 to March 2020 were identified as study population. The selection criteria for patients were: (1) age > 18 years; (2) the patients undergone surgical treatment for hip fracture; (3) the patient was willing to participate in this study and signed the written inform consent. The exclusion criteria were: (1) multiple fractures rather than only hip fracture; (2) pathological fractures caused by tumors; (3) the hip fractures more than 20 days from the time of injury; (4) patients with dementia or known mental illness; (5) patients with aphasia and hearing impairment; (6) Patients who did not sign informed consent or lost follow-up.

### Laboratory examination

All patients undergone preoperative laboratory examinations. The items of laboratory examinations included blood type, albumin, blood sugar, blood creatinine, urea nitrogen, thyroxine (T4), serum thyroid stimulating hormone (TSH), coagulation function, blood gas analysis, electrocardiogram, chest radiography, echocardiography and vascular ultrasound of both lower extremities.

### Delirium assessment

The diagnosis of delirium referred to the standards established by the the Diagnostic and Statistical Manual of Mental Disorders of the American Psychiatric Association, and we used the consciousness assessment method (CAM)[12] as a diagnostic tool. CAM included the following four aspects: (1) acute onset, mood changes; (2) inattention; (3) disordered thinking; (4) changes in consciousness level. If the patient has items 1 and 2 at the same time, plus either 3 or 4, then delirium can be diagnosed. All patients who met the inclusion criteria were divided into two groups according to whether postoperative delirium occurred: delirium group and non-delirium group.

### Outcome assessment and collection

The baseline collected information included the age, weight, height, time of injury, the complicated diseases such as hypertension, diabetes mellitus et al. All patients underwent surgery under general anesthesia or lumbar anesthesia, and postoperative intravenous analgesia (PCA) was used. Postoperative assessment was performed by an anesthesiologist and a geriatrician. At 4 pm on the first and second days after surgery, CAM were used to assess whether delirium occurred and its severity, and visual analog score (VAS) was used to evaluate the pain of rest and motion status.

### Statistical analysis

SPSS 22.0 statistical software was used for data analysis in this study, and t-test was used for the comparison of continuous data between two groups; chi-square test was used for the comparison of categorical data between two groups. The independent risk factors were screened out using logistic regression analysis, and we drew the receiver operating curve (ROC) and calculated the area under the curve (AUC) to analyze its predictive value. p < 0.05 was considered that the difference was statistically significant.

## Results

### The information of patients

A total of 568 postoperative patients with hip fracture were included, of which 82 patients had delirium and 486 patients did not have delirium. The incidence of delirium in postoperative patients with hip fracture was 14.44 %. As Table 1 presented, there were significant difference on the age, history of delirium between patients with delirium and without delirium (all *p* < 0.05). And there were significant differences on the gender, BMI, hypertension, diabetes mellitus, hyperlipidemia, type of fracture, preoperative ADL score and the method of anesthesia between two groups (all *p* > 0.05).

**Table 1:** The general information of delirium and non-delirium patients

Items	Delirium group(*n* = 82)	Non-delirium group(*n* = 486)	t/χ^2^	p
Age	79.5 ± 5.33	70.2 ± 5.08	15.374	0.009
Male/female	29/53	185/301	2.209	0.083
BMI (kg/m^2^)	22.3 ± 3.15	22.4 ± 2.93	1.812	0.066
Hypertension	43/39	260/226	1.174	0.125
Diabetes mellitus	22/60	125/361	1.390	0.097
Hyperlipidemia	13/69	79/407	3.357	0.866
History of delirium	38/44	41/445	1.194	0.031
Type of fracture			1.127	0.095
Femoral neck fracture	30	183		
Intertrochanteric fracture	51	299		
Subtrochanteric fracture	1	4		
Preoperative ADL score	95.4 ± 5.04	94.9 ± 5.18	17.316	0.230
Method of anesthesia			1.182	0.094
Lumbar anaesthesia	47	292		
General anaesthesia	35	194		

Notes: *BMI* body mass index. *ADL* ability of daily living score

The comparison of preoperative laboratory examination and postoperative VAS score.

As Table 2 presented, there were significant difference on the preoperative albumin, TSH and the rest score on the first postoperative day (all *p* < 0.05). There were no significant differences on the preoperative hemoglobin, blood sugar, creatinine, urea nitrogen, T3, T4, FT3, FT4, PaO_2_, PaCO_2_, the motion score on the first postoperative day and the VAS on the second postoperative day between two groups(all *p* > 0.05).

**Table 2:** The results of patients’ preoperative laboratory examination and postoperative VAS score

Items	Delirium group(*n* = 82)	Non-delirium group(*n* = 486)	t/χ^2^	p
Albumin(g/L)	37.25 ± 5.18	43.39 ± 4.96	8.098	0.035
Hemoglobin(g/L)	121.04 ± 14.37	121.18 ± 15.20	6.495	0.095
Blood sugar(mmol/L)	7.73 ± 1.29	7.69 ± 1.03	1.145	0.110
Creatinine(µmol/L)	77.29 ± 9.06	75.13 ± 9.15	8.440	0.127
Urea nitrogen(mmol/L)	7.80 ± 1.28	7.59 ± 1.17	1.096	0.093
T3(mU/L)	1.32 ± 0.11	1.33 ± 0.15	1.030	0.298
T4(mU/L)	95.18 ± 17.35	97.42 ± 16.55	9.194	0.166
TSH(mU/L)	1.65 ± 0.28	2.68 ± 0.77	0.926	0.032
FT3(mU/L)	3.01 ± 0.65	3.31 ± 0.64	1.118	0.057
FT4(mU/L)	16.39 ± 2.75	16.25 ± 2.18	1.093	0.056
PaO_2_(mmHg)	83.32 ± 19.06	84.18 ± 17.06	5.275	0.102
PaCO_2_(mmHg)	32.48 ± 3.18	33.24 ± 4.10	2.184	0.063
VAS on the first postoperative day				
Resting	2.01 ± 0.84	0.97 ± 0.41	1.106	0.032
Motion	4.28 ± 1.13	3.95 ± 1.02	1.081	0.084
VAS on the second postoperative day				
Resting	0.79 ± 0.27	0.69 ± 0.38	1.194	0.198
Motion	2.87 ± 0.95	2.85 ± 0.88	1.056	0.085

Notes:* PaO*_2_ partial pressure of oxygen; *PaCO*_2_ partial pressure of carbon dioxide; *T4* total thyroxine; *T3* triiodothyronine; *FT4* free thyroxine; *FT3 *free triiodothyronine; *VAS* visual analog score; *TSH* thyroid stimulating hormone

The risk factors for the post-operative delirium in patients with hip fracture.

As Table 3 showed, logistic regression analyses indicated that the preoperative albumin (OR 4.382, 2.501 ~ 5.538), history of delirium (OR 2.197, 1.094 ~ 3.253), TSH (OR1.245, 1.077 ~ 1.638), the resting score on the first postoperative day (OR1.235, 0.944 ~ 1.506) and age(OR1.185, 0.065 ~ 1.814) were the independent risk factors for the postoperative delirium in patients with hip fracture(all p < 0.05).

**Table 3:** Logistic regression analysis on the risk factors for the post-operative delirium in patients with hip fracture

Items	β	SE	OR	95 %CI	p
Albumin(g/L)	0.115	0.249	4.382	2.501 ~ 5.538	0.022
History of delirium	0.932	0.324	2.197	1.094 ~ 3.253	0.039
TSH (mU/L)	0.324	0.145	1.245	1.077 ~ 1.638	0.041
The resting score on the first postoperative day	0.695	0.394	1.239	0.944 ~ 1.506	0.016
Age	0.744	0.228	1.185	0.065 ~ 1.814	0.046

Notes: *TSH* thyroid stimulating hormone

The predictive value analysis.

**Fig. 1:**
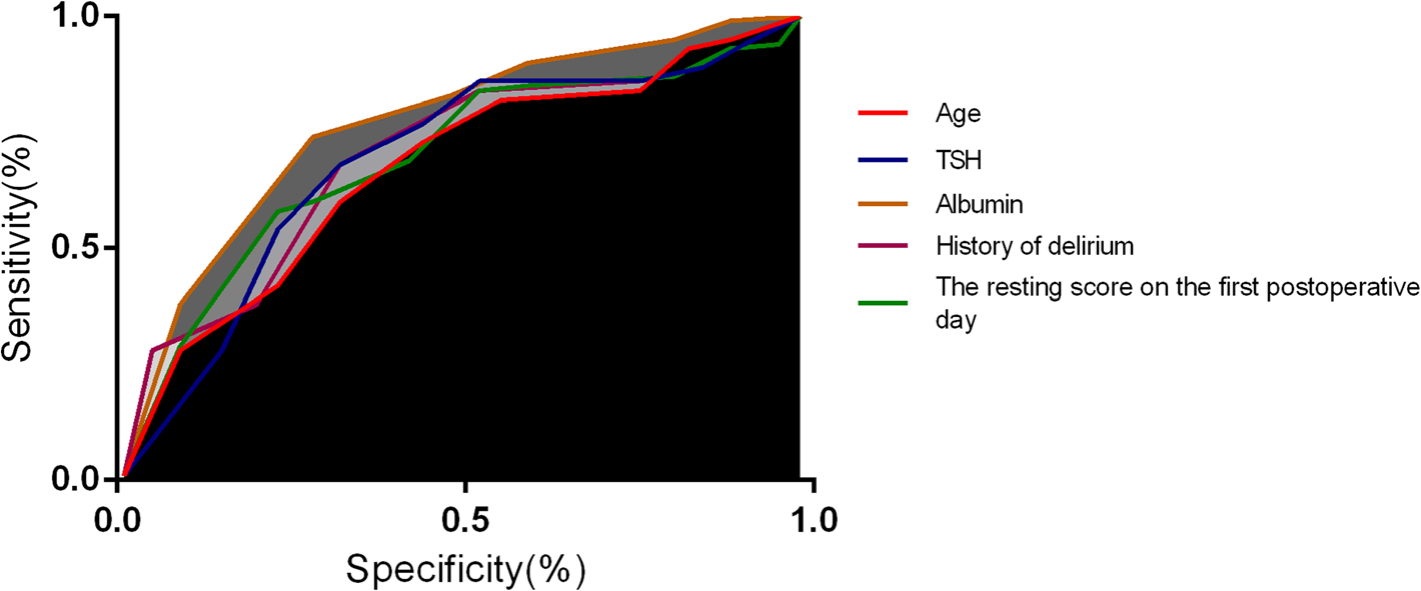
The ROC of the potential factors for the post-operative delirium in patients with hip fracture

Figure 1 presented the ROC of the potential factors for the post-operative delirium in patients with hip fracture. As indicated in Table 4, the AUC of albumin, history of delirium, TSH, the resting score on the first postoperative day and age were 0.794, 0.754, 0.746, 0.721 and 0.689 respectively.

**Table 4:** The predictive value of related factors

Variables	AUC	95 %CI	SEM
Albumin(g/L)	0.794	0.702–0.858	0.056
History of delirium	0.754	0.648–0.810	0.051
TSH (mU/L)	0.746	0.679–0.781	0.058
The resting score on the first postoperative day	0.721	0.658–0.790	0.057
Age	0.689	0.602–0.724	0.543

Notes: *TSH* thyroid stimulating hormone

## Discussion

Hip fractures seriously endanger the physical and mental health of the elderly and the quality of life[13]. The hip fractures are associated with inability to mobile and a series of complications such as decubitus ulcers, lung infections[14, 15]. At present, for hip fracture patients, surgical treatment, including fracture internal fixation and hip replacement, is the main and preferred method of treatment[16]. However, the surgical treatment itself can also lead to many postoperative complications. Delirium a is one of the most common and serious complications after surgery for hip fracture patients[17]. The pathogenesis of postoperative delirium is very complicated, and so far, it has not been clarified. In recent years, there are many emerging hypotheses, including the central neurotransmitter theory, the brain metabolic level change theory, surgical stress theory and neuroinflammation hypothesis et al[18–20]. In this present study, 82 of the 568 patients have postoperative delirium, the incidence of postoperative delirium is14.44 %. Previous study[21] has reported that the incidence of postoperative delirium in elderly patients with hip fracture is about 20.5 %, which is consistent with our findings. And we have found that preoperative albumin, history of delirium, TSH, the resting score on the first postoperative day and age were the independent risk factors for the postoperative delirium in patients with hip fracture. It is suggested that the above risk factors may be the focus of prevention and treatment of delirium during the perioperative period, and preventions and early warning should be performed with target on those risks.

Delirium is an acute brain dysfunction that manifests as distracted attention, declined recognition, disturbances of circadian clock, emotional and mental disorders[22]. Its incidence increases with age, and it can reach 14 % in people over 85 years of age[23]. In the surgical field, patients with hip fractures over 65 years of age have a higher incidence of postoperative delirium[24]. This study also confirmed that age is a risk factor for postoperative delirium. Previous studies[25–27] have shown that for every increased year of age, the incidence of delirium increases by 1.13 times. The hip fracture is generally seen in elderly patients, and elderly patients with multiple systemic diseases such as central nervous system degeneration, which are prone to nerve dysfunction after surgical stress. Therefore, the use of clustered perioperative strategies, such as accelerated surgical rehabilitation strategies, has important clinical significance for reducing the incidence of delirium[28].

It’s been reported that about 20–65 % of the elderly have different degrees of albumin deficiency[29, 30]. In addition to maintaining plasma osmotic pressure, binding with various ligands, as well as anticoagulation and maintaining acid-base balance, albumin also has the function of antioxidant, scavenging free radicals and protecting microcirculation[31]. Preoperative hypoalbuminemia directly affects the pharmacological effects of anesthetic drugs, resulting in uncertainty in the dose selection and duration of action, which can directly or indirectly affect postoperative neural function[32]. Consistent with previous reports[33, 34], this study also confirms that preoperative hypoproteinemia is associated with postoperative delirium. This result shows that actively correcting preoperative hypoproteinemia is of great significance for the prevention and treatment of postoperative delirium in patients with hip fracture.

Postoperative pain is closely related to the occurrence of delirium, and good postoperative analgesia is an important measure to prevent delirium[35]. The results of this study indicate that only resting VAS on the first postoperative day is an independent risk factor for delirium. As one of the important noxious stimuli, acute pain can activate a variety of neurotransmitter transmission pathways, produce a synergistic effect of inflammatory response and oxidative stress response, which may eventually lead to delirium[36]. Therefore, patients with hip fractures should pay attention to pain management and actively implement early multimodal analgesic strategies, such as the use of nerve block, local infiltration of wound anesthesia, etc. Besides, reasonable sedation should be implemented. For hospitals with monitoring conditions, using dexmedetomidine in small doses can relieve anxiety and exert its advantages in reducing delirium.

Studies[37, 38] on the correlation between subclinical thyroid dysfunction and dementia and cognitive dysfunction suggest that compared with normal thyroid function, patients with subclinical hyperthyroidism (TSH < 0.45 mU/L) has higher risk of cognitive dysfunction within the onset of 5 to 9 years, and subclinical hypothyroidism is not significantly related to cognitive dysfunction. Although there has been no report on the correlation between subclinical thyroid dysfunction and delirium, the results in this study have indicated that subclinical hyperthyroidism may be a risk factor for postoperative delirium. The potential reasons may be that hyperthyroidism can lead to neuronal necrosis and increased oxidative stress, and some target genes of thyroid hormone may be related to the coupling of neurons[39]. Besides, the occurrence of dementia is related to the reduction of choline in the brain[40]. Some studies[41, 42] have found that in patients with untreated hyperthyroidism, the ratio of choline/creatinine in the brain decreases, and it can return to normal after drug treatment. Therefore, TSH should be used as a routine screening indicator for patients with hip fracture.

The following limitations in this present study should be considered. Firstly, our study is a retrospective analysis with a small sample size, some biases may be existed, and it can be underpower to detect the potential risks. Secondly, some other potential risks such as time to surgery, estimated blood loss during the surgery have been reported to be associated with the postoperative delirium, we could not include those factors into analysis due to the lack of information. Further investigations are needed to identify the potential risks and treatment of postoperative delirium.

## Conclusions

In conclusion, the prevalence of postoperative delirium in hip fracture patients is relatively high. At the same time, preoperative albumin, history of delirium, TSH, the resting score on the first postoperative day and age were the independent risk factors for the postoperative delirium in patients with hip fracture. Clinicians should correctly assess the patient’s physical state and function of various organs before the operation, improve the patient’s nutritional status. However, given the small size of our study, it’s necessary to conduct more related researches to elucidate the prevention and management of deliriums in clinical settings.

## Data Availability

All data generated or analyzed during this study are included in this published article.

## Abbreviations

BMI: Body mass index
ADL: Ability of daily living
CAM: Consciousness assessment method
PCA: Postoperative intravenous analgesia
VAS: Visual analog score
ROC: Receiver operating curve
AUC: Area under the curve
PaO_2_: Partial pressure of oxygen
PaCO_2_: Partial pressure of carbon dioxide
T3: Triiodothyronine
T4: Total thyroxine
FT3: Free triiodothyronine
FT4: Free thyroxine
TSH: Thyroid stimulating hormone
